# Differential neutrophil chemotactic response towards IL-8 and bacterial N-formyl peptides in term newborn infants

**DOI:** 10.1080/03009734.2016.1228721

**Published:** 2016-10-03

**Authors:** Maria E. Stålhammar, Lena Douhan Håkansson, Anders Jonzon, Richard Sindelar

**Affiliations:** a Department of Women's and Children's Health, Uppsala University, Uppsala, Sweden;; b Department of Medical Sciences, Uppsala University, Uppsala, Sweden

**Keywords:** Chemotaxis, chemoattractants, fMLP, innate immunity, IL-8, neutrophils, newborn infants

## Abstract

**Background:**

A prerequisite for an effective innate immunity is the migrative ability of neutrophils to respond to inflammatory and infectious agents such as the intermediate interleukin (IL)-8 and the end-target formyl-methionyl-leucyl-phenylalanine (fMLP) chemoattractants. The aim was to study the chemotactic capacity of neutrophils from newborn infants and adults in response to IL-8 and the bacterial peptide fMLP.

**Methods:**

In the under-agarose cell migration assay, isolated leukocytes from healthy adults and from cord blood of healthy term newborn infants were studied with dose responses towards IL-8 and fMLP. The same number of leukocytes (1 × 10^5^ cells), with the same distribution of neutrophils and monocytes, were analyzed in neonates and adults. Chemotaxis was distinguished from randomly migrating neutrophils, and the neutrophil pattern of migration, i.e. the migration distance and the number of migrating neutrophils per distance, was evaluated.

**Results:**

In comparison to adults, fewer neutrophils from newborn infants migrated towards IL-8 and for a shorter distance (*P* < .01, respectively). The number of neutrophils migrating to different gradients of fMLP, the distance they migrated, and the correlation between the number and the distance were the same for neonates and adults. Random migration did not differ in any instance.

**Conclusion:**

Chemotaxis of neutrophils from newborn infants was as co-ordinated as neutrophils from adults in response to fMLP, whereas the response to IL-8 was reduced. The differential response of neutrophils from neonates to intermediate and end-target chemoattractants could indicate a reduced infectious response.

## Introduction

Neutrophils are the most abundant cells of the innate immune system. They are one of the first inflammatory cells to migrate towards the site of invading micro-organisms. Upon stimuli the cells migrate from the blood into the tissue by rolling, adhesion, and transmigration through the endothelium, and finally by graded chemotaxis to the site of the invading bacteria, where the neutrophils phagocyte the invading microbes (1).

The immune system of term (born between 37 and 42 weeks’ gestational age) newborn infants gives a certain protection against pathogens but, due to an immature immunologic response, puts them at risk of developing severe sepsis during the neonatal period with increased risk of morbidity and mortality (2). A reduced capacity to increase neutrophil production (3), together with an observed decreased chemotaxis into infected sites (4,5), makes the newborn infant vulnerable to bacterial infections. Because of a delayed neutrophil influx at the site of infection, increased bacterial growth can lead to rapidly progressive infection which in turn may deplete the limited bone marrow neutrophil pool (3).

Chemoattractants can be divided into end-target chemoattractants and intermediary endogenous chemoattractants based on their intracellular hierarchy, with a neutrophil preference towards end-target chemoattractants such as formyl-methionyl-leucyl-phenylalanine (fMLP) and complement component 5a (C5a) over intermediary chemoattractants such as interleukin (IL)-8 and leukotriene b4 (LTB4), even in the presence of higher concentrations of the latter (6–8). The endogenous intermediate chemotactic factor IL-8 is produced by inflammatory cells (9), and the end-target chemotactic peptide fMLP is generated by micro-organisms like *Escherichia coli* and *Staphylococcus aureus* (10,11). Although many different chemoattractants direct cell migration they all bind to the same G protein-coupled receptor family (GPCR) (12). Chemotaxis involves a cascade of events: formation of signaling pathways, receptor polarization, adhesion receptor activation, and cytoskeletal reorganization (13).

*In vitro* studies of chemotaxis can supply us with information on the inflammatory capacity of individual patients. The under-agarose migration assay is a method to study graded chemotactic leukocytes and has been used for determining the effects of diseases on leukocyte chemotaxis (14), the role of several signaling pathways, and the role of adhesion in neutrophil chemotaxis (6,13). With the under-agarose assay it is possible to separate the number of chemotactic cells from cells moving randomly, i.e. through chemokinesis (7,13,15), which is an advantage when comparing different groups of patients. The number of migrating cells, together with their migration distance, reveals the migration pattern of different neutrophil populations (6,7,13,16). The under-agarose assay is unique in the way that it includes the possibility to generate multiple chemotactic gradients in different spatial and temporal combinations (13,17). Real-time observations of cellular movement and migrating distance are also facilitated with the agarose assay, as the migrating cells are exposed to a resistance in a gradient of chemoattractants, whereby the number of migrating cells are limited and more easily counted (17).

The aim was to study the chemotactic capacity of neutrophils from term newborn infants as compared to adults, in response to the intermediate inflammatory factor IL-8 and the bacterial end-target peptide fMLP with the under-agarose assay. The hypothesis was that neutrophils from newborn infants are less chemotactic than neutrophils from adults, in this graded chemotactic assay, and are thereby more vulnerable to infectious insult. In order to define the pattern of migration of the neutrophil populations, we determined the correlation between the number of migrating neutrophils and the distance they migrated towards different gradients of IL-8 and fMLP. Determining the response to intermediate and end-target chemoattractants enabled us to distinguish at what stage of bacterial infection the reduced chemotactic response of neutrophils from neonates might occur.

## Material and methods

### Subjects and blood analysis

Healthy, term, newborn infants (*n* = 8) born at 38–39 weeks’ gestational age with a weight of 2,800–4,500 g and a mean Apgar score of 8 at one minute of age, 10 at five minutes, and 10 at ten minutes were included in the study. Blood was collected from the placental side of the umbilical cord immediately after elective cesarean section was performed under spinal anesthesia. Since neutrophil activity is increased due to fetal stress in newborn infants during vaginal delivery (18,19), we studied neutrophils from the umbilical cord of healthy term newborn infants, immediately after elective cesarean section. Peripheral blood was collected from healthy adults (*n* = 8), aged 18–65 years old. All blood samples (from newborn infants and adults) were collected in sodium heparin tubes (Vacuette, Hettich Lab instrument AB, Sollentuna, Sweden). In all experiments one newborn infant and one adult participated simultaneously.

### Preparation of leukocytes

Leukocytes from newborn infants and adults were isolated from heparinized blood by means of dextran sedimentation (20), and contained 65%–75% neutrophils with no difference in distribution between neonates and adults. Leukocytes were then suspended in buffer to the remaining concentration of 1 × 10^7^ cells/mL. The final number of leukocytes in each cell well was 1 × 10^5^ cells.

### Preparation of chemoattractants

IL-8 (Life Technologies, Carlsbad CA, USA) and fMLP (Sigma-Aldrich, St Louis, MO, USA) were used as stimuli. The chemoattractants were serially diluted in buffer consisting of 53% RPMI 1640 (Gibco by Life Technologies, Carlsbad, CA, USA), 13% hyclone fetal bovine serum (FBS, Nordic Biolabs, Täby, Sweden), and sodium bicarbonate (NaHCO_3_, Sigma-Aldrich) mixed with 33% Hanks balanced salt solution (HBSS, Gibco by Life Technologies). IL-8 was used at concentrations of 0.1 and 1 μM and fMLP at concentrations of 0.01, 0.1, and 1 μM.

### The under-agarose cell migration assay

**Figure 1.**
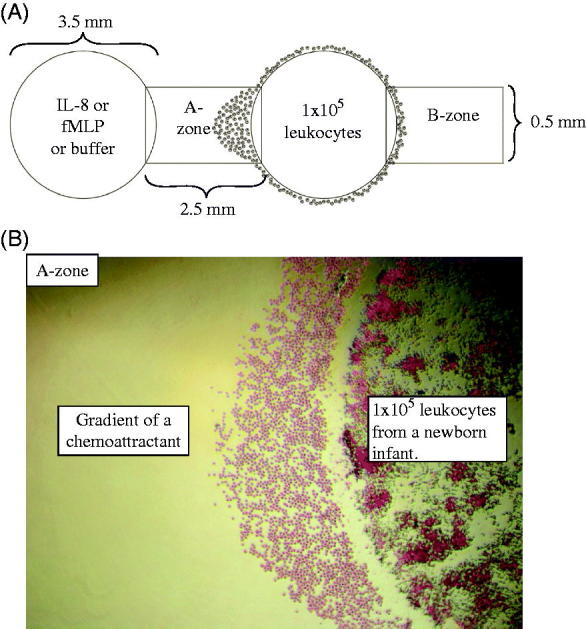
A: Schematic representation of the under-agarose migration assay. Wells are 3.5 mm wide and 2.5 mm apart. The two outer wells were loaded with 10 μL of 10^7^ leukocytes/mL from a newborn infant or an adult (1 × 10^5^ leukocytes/well). The central wells were loaded with IL-8 or fMLP or buffer (negative controls). By subtracting the number of neutrophils that migrated into the B-zone from the number in the A-zone, the number of neutrophils undergoing chemotaxis can be determined. B: An example of the migrating cells in the A-zone with 4× magnification, towards gradients of a chemoattractant. Note that only neutrophils have migrated into the A-zone at this time point.

The assay was performed according to the principles outlined by Heit and Kubes (13,15). Agarose gels were cast on tissue culture plates (Sigma-Aldrich) and stored overnight at 4 °C to make the gels firm and facilitate punching of the wells. Wells were punched the following day at a standardized distance of 2.5 mm from each other (Figure 1). Leukocytes from newborn infants and adults were added simultaneously to different tissue culture plates for each experiment. Leukocytes (1 × 10^5^ per well) were added to each of the two outer wells (Figure 1) in order to demonstrate that the migration was directed towards the gradient of the central well. Chemoattractant or buffer was added to central wells. Gels were incubated for 2 hours at 37 °C, 5% CO_2_, whereby only neutrophils migrated outside the wells. Methanol (100%) was added to each well to terminate the migration and fix the cells. The gels were stored overnight at 4 °C.

### Analysis of neutrophil chemotactic response

The gels were removed and the cells were stained with Wright’s stain (Sigma-Aldrich). Photos of the migration were taken with an inverted phase contrast microscope (Nikon Diaphot 300). Chemotactic neutrophils were distinguished histologically by their size and segmented nucleus. Only neutrophils were detected outside the wells, and they were counted in the predefined target zones A and B with a width of 0.5 mm (Figure 1(A,B)). Randomly migrating neutrophils appear in equal numbers in target zone A and B, while both chemotactic and randomly migrating cells are found in the A-zone (Figure 1(A,B)). The number of chemotactic neutrophils was thereby calculated by subtracting the number of cells in the B-zone from cells in the A-zone. All experiments were carried out simultaneously in adults and neonates, with three adults and three neonates during testing with IL-8, and five adults and five neonates during testing with fMLP. No patients or experiments were excluded.

### Migration distance and correlation to the number of migrating cells

The distance the neutrophils migrated under the agarose was measured from the edge of the well to the edge of the leading front (inside the A-zone). The correlation between the distance the cells migrated to gradients of IL-8 or fMLP and the number of migrating neutrophils in the A-zone was measured in order to define the pattern of migration of the neutrophil population.

### Statistical analysis

All statistical analyses were performed using IBM SPSS Statistics 20. All comparisons between newborn infants and adults, between different concentrations of IL-8, fMLP, and random migration, and the distance the cells migrated were made with Mann–Whitney *U* test for independent samples. A *P* value of ≤.05 was considered significant.

### Ethical approval

The study was approved 3 October 2007 by the Central Ethical Review Board (registration number Ups 03-692) at Uppsala University, Uppsala, Sweden. Written informed consent was obtained from the parents of all newborn infants as well as from the adults, before recruitment.

## Results

### Dose response

**Figure 2.**
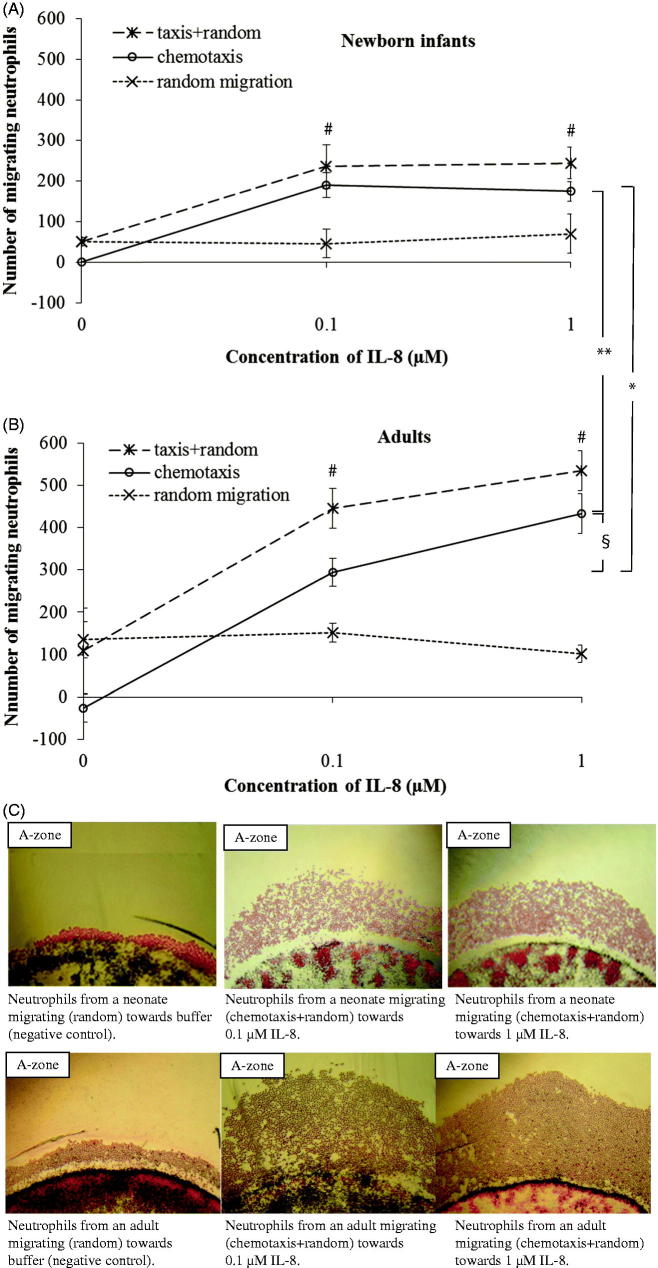
Dose response curves of migrating neutrophils to gradients of IL-8. Leukocytes from newborn infants (A) (*n* = 3) and adults (B) (*n* = 3) were exposed to different concentrations of IL-8 (0.1 μM and 1 μM) and buffer. Results are presented as mean ± SEM, and chemotaxis (taxis) was distinguished from random migration (random). C: Example of different patterns of neutrophil migration with 4× magnification, towards buffer (negative control), and gradients of IL-8 in one neonate and one adult. *P < .05 indicates significant difference between neonates and adults at 0.1 μM IL-8, and **P < .001 at 1 μM IL-8. §*P* < .05 indicates significant difference between 0.1 μM and 1 μM IL-8 in adults. At all concentrations of IL-8, a significantly higher number of neutrophils from both neonates and adults migrated towards IL-8 than to buffer (#*P* < .001). Note: ‘random migration’ counted from B-zone; ‘taxis + random’ counted from A-zone; ‘chemotaxis’ calculated from A-zone minus B-zone.

**Figure 3.**
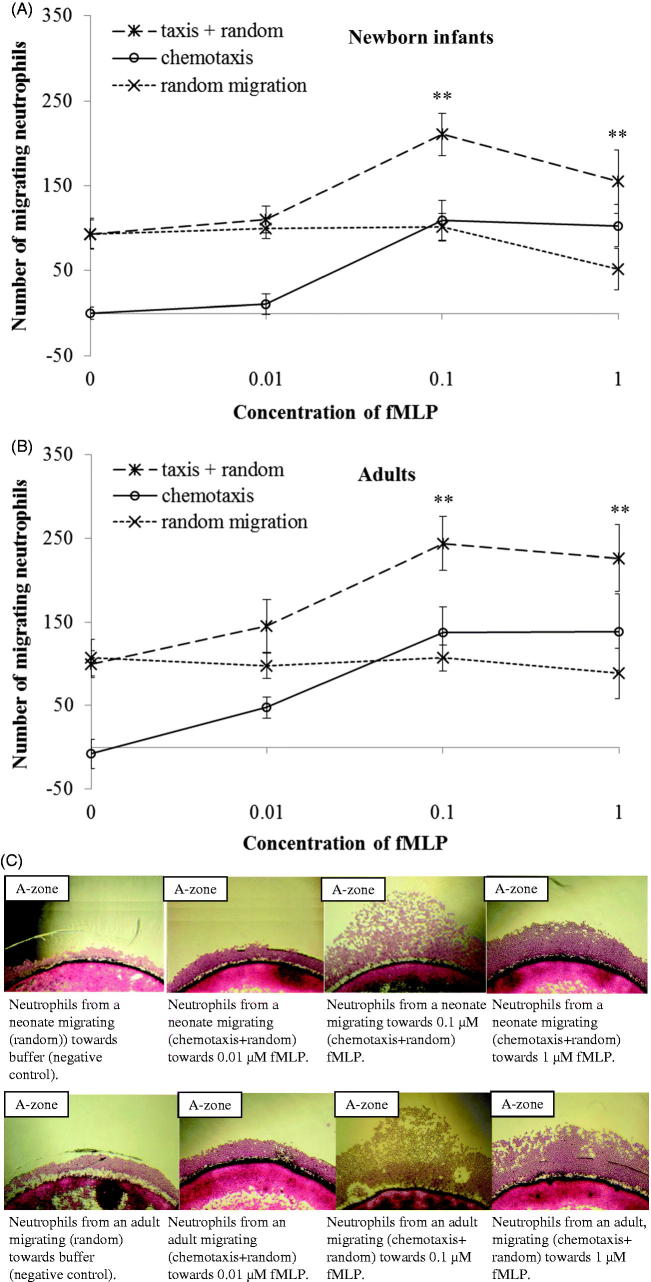
Dose response curves of migrating neutrophils to gradients of fMLP. Leukocytes from newborn infants (A) (*n* = 5) and adults (B) (*n* = 5) were exposed to different concentrations of fMLP (0.01 μM, 0.1 μM, and 1 μM) and buffer. Results are presented as mean ± SEM, and chemotaxis (taxis) was distinguished from random migration (random). C: Example of different patterns of neutrophil migration with 4× magnification, towards buffer (negative control), and gradients of fMLP in one neonate and one adult. At all concentrations of fMLP, a significantly higher number of neutrophils from both neonates and adults migrated towards fMLP than to buffer (**P < .01). Note: ‘random migration’ counted from B-zone; ‘taxis + random’ counted from A-zone; ‘chemotaxis’ calculated from A-zone minus B-zone.

In comparison to adults, a lower number of neutrophils from newborn infants migrated chemotactically towards IL-8 (*P* < .05; Figure 2). The number of chemotactic neutrophils migrating to different gradients of fMLP was the same in neonates as in adults (Figure 3). A significant chemotactic response was detected at concentrations of ≥0.1 μM IL-8 and ≥0.1 μM fMLP, both in newborn infants and adults (*P* < .001 and *P* < .01, respectively; Figures 2(A,B) and 3(A,B)). A non-saturated chemotactic response towards IL-8 (*P* < .05) was observed in adults compared to the saturated response at 0.1 μM IL-8 in neonates. For fMLP, saturated chemotactic responses were observed both in newborn infants and adults, and at the same concentration (0.1 μM fMLP; Figures 2(A,B) and 3(A,B)). The number of randomly moving neutrophils was the same in neonates and adults, and did not change with increasing concentrations of the chemoattractants (Figures 2 and 3).

### Migration distance

**Figure 4.**
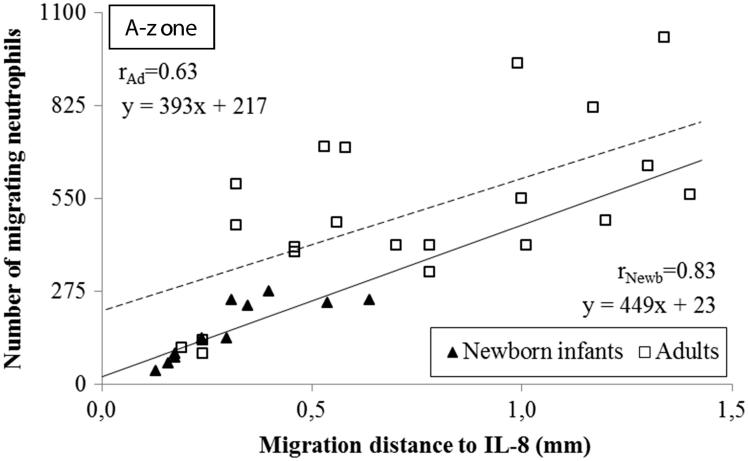
Correlation of the distance and the number of neutrophils migrating to different gradients of IL-8 in the A-zone. The distance the cells migrated was measured from the edge of the well to the edge of the leading cell front. The correlation coefficients in newborn infants was *r* = 0.83 compared to *r* = 0.63 in adults (*n* = 3; *P* < .001, respectively). Note the higher incipient migration at *x* = 0 and the longer distance of migration in adults compared to neonates.

**Figure 5.**
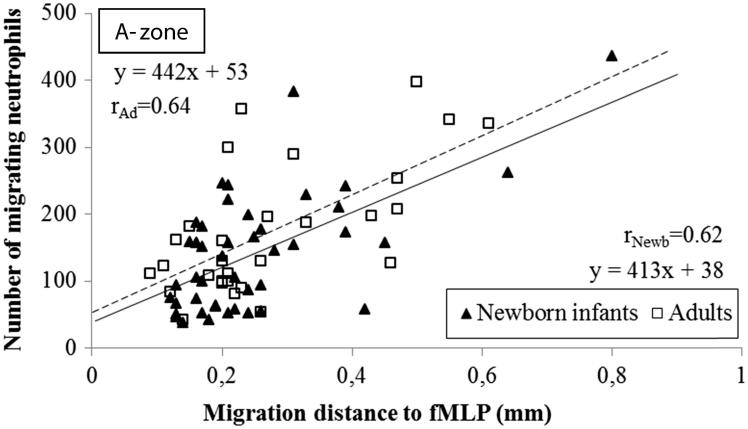
Correlation of the distance and the number of neutrophils migrating to different gradients of fMLP in the A-zone. The distance the cells migrated was measured from the edge of the well to the edge of the leading cell front. The correlation coefficients in newborn infants was *r* = 0.62 and *r* = 0.64 in adults (*n* = 5; *P* < .001, respectively). Note the comparable incipient migration and the equidistant migration in neonates and adults.

Neutrophils from newborn infants migrated a shorter distance towards both concentrations of IL-8 compared to neutrophils from adults (*P* < .01 and *P* < .001, respectively; Table 1, Figure 4). In contrast, chemotactic migration was equidistant for neutrophils from neonates compared to neutrophils from adults towards all concentrations of fMLP (Table 1, Figure 5).

**Table 1.** Migration distance (mean ± SEM) of chemotactic neutrophils towards different gradients of IL-8 and fMLP.

		Migration distance (μm)		
Chemoattractant	Concentration	Newborn infants	Adults	*P* value
IL-8	0	145 ± 15	223 ± 18	NS
IL-8	0.1 μM	221 ± 30	724 ± 155	<.01
IL-8	1 μM	412 ± 61	890 ± 130	<.001
fMLP	0	167 ± 13	215 ± 24	NS
fMLP	0.01 μM	186 ± 10	189 ± 16	NS
fMLP	0.1 μM	339 ± 42	359 ± 59	NS
fMLP	1 μM	270 ± 20	373 ± 82	NS

### Correlation between number of neutrophils and migration distance

There was a strong positive linear correlation between the number of migrating neutrophils and the distance they migrated towards IL-8 and fMLP in both neonates and adults, i.e. homogeneously migrating populations of neutrophils in all instances (Figures 4 and 5). The populations of neutrophils from newborn infants and adults were distinct in response to IL-8, with few cells migrating a shorter distance in neonates compared to many cells migrating a longer distance in adults (Figure 4). No such distinction could be made between neonates and adults with respect to the cells migrating towards fMLP, in terms of number of cells or migration distance (Figure 5). Deduced from the linear correlations, a markedly lower incipient migration to IL-8 was observed in newborn infants compared to adults, i.e.:

1. if *x* = 0 (the distance from the well), then *y* = incipient migration
  From which follows that the incipient migration for neonates versus adults are:
2. *y_neonates_* = 449 × 0 + 23 = 23 versus *y_adults_* = 393 × 0 + 217 = 217
3. *y_neonates_* = 23 versus *y_adults_* = 217

The ensuing migration (the slope of the linear correlation) towards IL-8 was the same in neonates and adults, as the slope of the linear correlations in neonates paralleled the slope in adults (449*x* versus 393*x*; Figure 4). Both the incipient and ensuing migration towards fMLP were similar in neonates and adults (Figure 5).

## Discussion

We observed that neutrophils from newborn infants have a differentiated chemotactic response towards intermediate and end-target chemoattractants. With the under-agarose migration assay we demonstrated that neutrophils from newborn infants are as co-ordinated as neutrophils from adults in response to fMLP. However, IL-8 induced a markedly lower migratory response in neutrophils from newborns than from adults in terms of the number of migrating cells and the distance of migration.

The protective response of the innate immune system to microbial or other agents is characterized by the accumulation of leukocytes in the affected tissue. The first activated leukocytes to respond are neutrophils, and their accumulation is preceded by endothelial cell activation, neutrophil to endothelial adherence interaction, and transmigration through the endothelium. These events are followed by a graded chemotactic migration along increased concentrations of chemoattractants in the tissue, and finally the release of reactive oxidants and other antimicrobial agents at the site of infection/inflammation. Migration in newborn infants has previously been studied with filter methods that mainly simulate the transmigration from blood through endothelium (4,21,22), whereas graded chemotactic migration in tissue has been paid less attention. The filter methods neither display the migration pattern nor the stimulated random migration, as non-stimulated and stimulated random migration cannot be separated by this method.

Since neutrophil activity is increased by fetal stress in newborn infants born by vaginal delivery (18,19), we studied only leukocytes from healthy term newborn infants delivered by cesarean section. Another factor we wanted to decrease was the release of IL-8, previously observed to be increased in newborn infants during vaginal delivery (19).

In the present study, the under-agarose cell migration assay gave us the possibility to assess graded chemotactic response with respect to the number of migrating neutrophils in relation to the migration distance, during different concentrations of chemoattractants, i.e. dose response, but also display the simultaneous stimulated chemokinesis and chemotaxis.

Previous studies have shown that neutrophils from neonates interact less with endothelial cells, compared to neutrophils from adults, and as a result have a reduced transmigration to a variety of different chemoattractants including IL-8 (23). This suggests a reduced migratory capacity of neutrophils in newborn infants and thereby a reduced antimicrobial defense. The finding in our study, that neutrophils from newborn infants have the same chemotactic capacity to respond to fMLP compared to neutrophils from adults, indicates that neutrophils from neonates are as capable of responding to this end-target bacterial chemoattractant, an observation supported by a recently published study on newborn infants made with a filter method and isolated leukocytes (22). The number and affinity of receptors for fMLP has also been reported to be the same in neutrophils from neonates and adults in several studies (24,25). Even though the IL-8Rs and the formyl peptide receptors belong to the same G protein-coupled receptor (GPCR) family, they stimulate different intracellular pathways that can affect chemotaxis, respiratory burst, and other cell activities differently (26,27). End-target attractants mediate chemotaxis via a p38 mitogen-activated protein kinase (MAPK) pathway, while intermediary attractants signal through phosphoinositide 3-kinase (PI3K)/Akt (6). These two pathways counteract each other, where the p38 MAPK pathway dominates by inhibiting the PI3K/Akt pathway, i.e. prior stimuli with fMLP reduces the sensitivity to IL-8 stimulation (6). Increased concentrations of lipopolysaccharides and fMLP in the blood has also been shown with *in vitro* studies to inhibit chemotaxis towards intermediate attractants such as IL-8, by down-regulating or desensitizing the IL-8 receptor by receptor phosphorylation, resulting in a possible reduced influx of neutrophils to sites of infection (8,28).

During graded chemotaxis, the neutrophils adapt to higher concentrations of the chemoattractant, thereby requiring a higher dose of stimulation in order to continue responding chemotactically as the cells become less sensitive to the same concentration of the stimulus (7). At higher concentrations of chemoattractant, chemotaxis arrests because the surface receptors of the neutrophils have become saturated (16). In our study of dose response, saturated chemotactic migration of the neutrophils was observed towards both IL-8 and fMLP in neonates, but only towards fMLP in adults, with the concentrations studied. It is not excluded that higher concentrations of IL-8 would eventually saturate the chemotactic migration in adults as well, as reported by others (13). Interestingly, studies of opposing chemotactic gradients on isolated neutrophils from adults have shown that end-target chemoattractants (fMLP, C5a) dominate over the intermediate chemoattractants (IL-8, LTB4), both in terms of the number and the distance of migrating neutrophils, irrespective of the concentrations of the applied chemoattractants (6,16). In our evaluation of both the number and the distance of migrating cells, the comparable chemotactic neutrophil response to fMLP in neonates and adults suggests that the end-target regulation is present in the newborn infant.

In order to exclude the possibility of a non-homogeneous neutrophil response, the correlation between the distance and the number of migrating neutrophils was calculated and was strong both in neonates and in adults, revealing that the migration of the neonate and adult populations was well assembled towards both IL-8 and fMLP. The incipient neutrophil migration towards the endogenously produced inflammatory factor IL-8 was lower in neonates compared to adults, and as the resulting chemotactic response was also reduced in neonates this could imply that neonates are less prepared for initiating an inflammatory response, an observation that needs to be further investigated.

We conclude that the neutrophil chemotactic response towards fMLP is as co-ordinated in neonates as in adults, whereas the response to IL-8 is lower in neonates, indicating that neutrophils from newborn infants have a differential response to end-target and intermediate chemoattractants, which could lead to a reduced response to infections in newborn infants.
